# MicroRNA Deregulation in Anaplastic Thyroid Cancer Biology

**DOI:** 10.1155/2014/743450

**Published:** 2014-08-19

**Authors:** Cesar Seigi Fuziwara, Edna Teruko Kimura

**Affiliations:** Department of Cell and Developmental Biology, Institute of Biomedical Sciences, University of São Paulo, Avenida Professor Lineu Prestes 1524, Room 414, CEP, Butantã, 05508-000 São Paulo, SP, Brazil

## Abstract

Anaplastic thyroid cancer (ATC) is among the most lethal types of cancers, characterized as a fast-growing and highly invasive thyroid tumor that is unresponsive to surgery and radioiodine, blunting therapeutic efficacy. Classically, genetic alterations in tumor suppressor *TP53* are frequent, and cumulative alterations in different signaling pathways, such as MAPK and PI3K, are detected in ATC. Recently, deregulation in microRNAs (miRNAs), a class of small endogenous RNAs that regulate protein expression, has been implicated in tumorigenesis and cancer progression. Deregulation of miRNA expression is detected in thyroid cancer. Upregulation of miRNAs, such as *miR-146b*, *miR-221*, and *miR-222*, is observed in ATC and also in differentiated thyroid cancer (papillary and follicular), indicating that these miRNAs' overexpression is essential in maintaining tumorigenesis. However, specific miRNAs are downregulated in ATC, such as those of the *miR-200* and *miR-30* families, which are important negative regulators of cell migration, invasion, and epithelial-to-mesenchymal transition (EMT), processes that are overactivated in ATC. Therefore, molecular interference to restore the expression of tumor suppressor miRNAs, or to blunt overexpressed oncogenic miRNAs, is a promising therapeutic approach to ameliorate the treatment of ATC. In this review, we will explore the importance of miRNA deregulation for ATC cell biology.

## 1. Introduction

Anaplastic thyroid cancer (ATC) is the most lethal histotype of thyroid cancer, responsible for more than one-third of thyroid cancer-related deaths [1]. The fast-growing nature of this type of cancer and its refractoriness to radioiodine treatment due to tumors not concentrating iodine limit the efficacy of therapeutic interventions [2]. Thus, ATC patients display a rapidly progressive disease that may cause death within six months [3].

ATC's clinical pathology is characterized by the aggressive behavior of cancer cells, which results in a rapid enlargement of the neck tumor to invade adjacent tissue and migrate as distant metastases, usually with secondary sites in the lungs, bone, and brain [4]. During this process, activation of epithelial-to-mesenchymal transition (EMT) is a key feature of anaplastic transformation. Moreover, loss of expression of differentiation markers (iodine metabolizing genes) and, consequently, loss of iodine uptake are markers of this process that negatively impact on ATC radioiodine responsiveness.

Unlike the differentiated histotypes of thyroid cancer, papillary thyroid cancer (PTC) and follicular thyroid cancer (FTC) that show a single driver mutation pattern, the tumorigenesis of ATC is not completely understood: it may arise* de novo* or from a preexisting well-differentiated cancer (PTC/FTC). The most prevalent genetic alterations in ATC are mutations in the* TP53* gene at codon 273 as observed in more than 70% of ATC samples [5, 6], leading to p53 loss of function. Moreover, mutations in the telomerase gene (*TERT*) are frequently seen in aggressive thyroid cancers, including in poorly differentiated and anaplastic thyroid cancers [7, 8], and lead to increased transcriptional activity of TERT. However, additional mutations in MAPK signaling (RAS and BRAF genes), in PI3K signaling (PIKCA and PTEN), and in Wnt signaling (*β*-catenin and APC) have also been detected in ATC [9–12].

Animal models have contributed to an understanding of the molecular transformation of ATC. The thyroid cancer progression hypothesis is corroborated by a transgenic mouse model for the early activation of the* BRAF* ^*T1799A*^ oncogene restricted to the thyroid gland (Tg-BRAF) that is affected by high penetrance PTC that undergoes temporal dedifferentiation [13]. Molecular analysis of Tg-BRAF mice-derived tumors reveals the deregulation of TGF*β* signaling upon prolonged stimulation of the BRAF oncogene, with enhanced TGF*β* signaling transduction and a shift to EMT by ZEB1 and ZEB2 transcription factor activation [14]. Another transgenic model with BRAF activation in adult mice also gives rise to differentiated thyroid cancer; however, progression to ATC requires* TP53* gene deletion [15], corroborating the multistep cancer progression hypothesis for ATC. A mouse model harboring defective PI3K signaling gives rise to aggressive thyroid cancer. Transgenic mice carrying deletions of* Pten* and* TP53* [*Pten*,* p53*]^(thyr−/−)^ develop ATC with loss of expression of the thyroid transcription factors,* Nkx2-1*,* Pax-8*, and* Foxe1*, and thyroid differentiation markers,* Nis*,* Tpo*,* Tg*,* Tshr*, and* Duox* [16]. [*Pten*,* p53*]^(thyr−/−)^ mice thyroid tumors undergo TGF*β* signaling induced EMT and demonstrate increased pSMAD2 and vimentin levels, but loss of E-cadherin expression.

**Figure 1:**
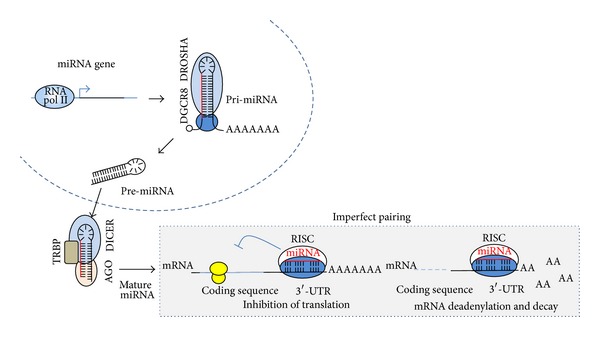
Biogenesis of miRNA. Transcription of miRNA by RNA polymerase II yields a long primary transcript (pri-miRNA) that contains a cap 5′ and poly-A tail. The complex DROSHA/DGCR8 cleaves pri-miRNA and gives rise to an miRNA precursor (pre-miRNA) that is exported to the cytoplasm and further processed by DICER endonuclease. An miRNA duplex associates with the RISC complex and retains the mature strand of miRNA. This complex directs imperfect binding to 3′-UTR region of target mRNA, leading to a reduction in protein levels via translation blockage and mRNA deadenylation and decay.

Recently, a pivotal role for microRNAs (miRNAs) in cancer has emerged with increasing evidence showing that they may drive and potentiate oncogenesis. The miRNAs are a class of small noncoding RNAs (~20 nt) that regulates posttranscriptional gene expression. These miRNAs are transcribed as long primary RNAs that undergo sequential cleavages by Drosha and Dicer ribonucleases in the nucleus and cytoplasm, respectively, to yield mature miRNA. In turn, mature miRNA associates with the protein complex RISC (RNA-induced silencing complex), which directs pairing with the 3′-UTR region of target mRNAs. Imperfect pairing leads to translational blockage of target mRNA and, also, mRNA deadenylation and decay (Figure 1).

**Figure 2:**
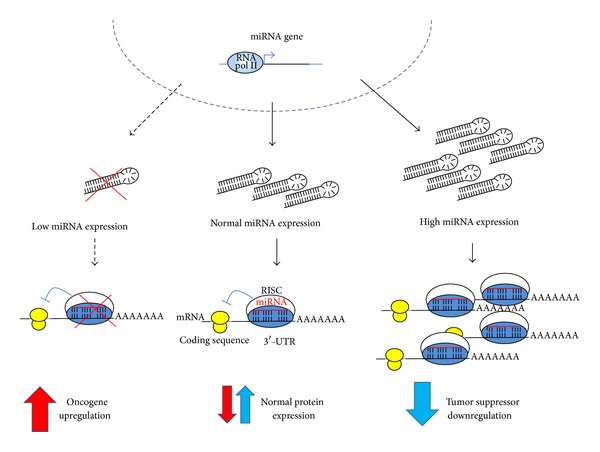
Some miRNAs act as oncomiRs. Deregulation of miRNA changes physiological protein level balances and enhances the oncogenic process where (1) low expression of an miRNA may enhance protein translation of an oncogenic protein or (2) high expression of an miRNA may repress the translation of a tumor suppressor gene. Both situations may occur concomitantly in cancer as observed in anaplastic thyroid cancer.

Essentially, miRNA deregulation in cancer is oncogenic (oncomiR) under two different conditions: (a) when overexpressed, miRNAs may block tumor suppressor gene translation or (b) when underexpressed, miRNAs may derepress protooncogene mRNA translation (Figure 2).

**Figure 3:**
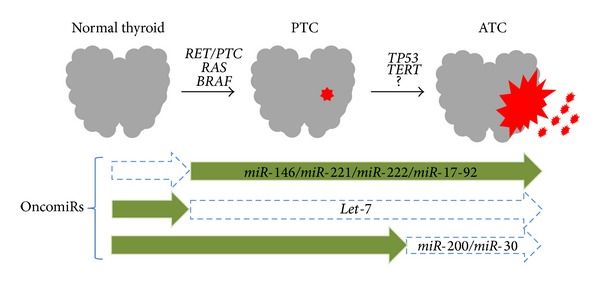
Thyroid oncogenesis and miRNAs. Activation of MAPK oncogenes by mutations or rearrangements leads to PTC. Progression to ATC may be associated with the acquisition of additional genetic alterations such as* TP53* mutations. Deregulation of miRNA occurs during thyroid oncogenesis, with specific upregulation of miRNAs such as* miR-146*,* miR-221*,* miR-222*, and* miR-17-92* cluster, and loss of* let-7* expression, in both PTC and ATC. Exclusive downregulation of miRNAs, such as* miR-200* and* miR-30*, is observed in ATC.

Deregulation of miRNA in thyroid cancer was initially described by He et al. in a group of PTCs [17]. The same group of upregulated miRNAs, such as* miR-146b*,* miR-221*, and* miR-222*, is commonly detected in ATC and in differentiated thyroid cancers (papillary and follicular histotypes) [18] and also in a fraction of benign nodules [17, 19], indicating that persistent expression of these miRNAs may be necessary to maintain tumorigenesis. However, ATC also shows exclusive reduction of certain miRNAs with tumor suppressor properties, indicating a role for these miRNAs in tumor aggressiveness (Figure 3). Investigating tumour samples from a group of ten ATC patients by microarray analysis, the seminal study of Visone et al. [20] revealed the repression of* miR-30d*,* miR-125b*,* miR-26a*, and* miR-30a-5p*. In addition, another study showed a reduction of several members of the* let-7* and* miR-200* family of miRNAs [21] and overexpression of* miR-221*,* miR-222*, and* miR-125a-3p*.

In this review, we will explore some aspects of the deregulated miRNA found in anaplastic thyroid cancer to address the molecular biology and signaling pathways implicated in the clinical-pathological characteristics of this type of cancer (Table 1).

## 2. Downregulated miRNAs in ATC

Specific miRNAs are reduced in thyroid cancer, such as the* let-7* family, but other miRNAs, such as the* miR-200* and* miR-30* families, are exclusively downregulated in ATC, indicating that the latter miRNAs may play a role in the acquisition of more aggressive tumor characteristics (i.e., enhanced cell invasion and migration).

### 2.1. *miR-200* Family

The* miR-200* family is composed of the* miR-200a*,* miR-200b*, and* miR-200c* genes, which are usually downregulated in ATC [21]. The* miR-200a* and* miR-200b* genes make up a cluster located on chromosome 1, while the* miR-200c* gene is located on chromosome 12. An important transcriptional activator of* miR-200* is p53, which binds to the promoter region of* miR-200c* at multiple sites. Interestingly, a* TP53* mutation at a DNA binding domain as present in ATC impairs downstream transcriptional activation of* miR-200* [22]. Moreover, p53 is an important controller of tumor suppressor miRNAs, such as those of the* miR-34* family, and also influences miRNA processing [23], besides having a classical role in DNA repair and genomic stability. Another signaling pathway that influences* miR-200* expression is the EGF pathway. Overexpression of the EGF receptor, EGFR, is observed in ATC [24], while EGFR knockdown in ATC cells restores* miR-200* expression and represses the expression of mesenchymal markers [25]. Classically, TGF*β* signaling induces epithelial-to-mesenchymal transition (EMT), via the transcriptional activation of ZEB1 and ZEB2 [21], inducing a mesenchymal phenotype, with the expression of vimentin and repression of E-cadherin as observed in ATC. Interestingly, the* miR-200* family is an important regulator of the EMT process by regulating ZEB1 and ZEB2 protein levels. Downregulation of* miR-200* in ATC would potentiate the TGF*β*-mediated EMT switch and enhance aggressiveness.

### 2.2. *miR-30* Family

The* miR-30* family of tumor suppressor miRNAs is composed of five members:* miR-30a*,* miR-30b*,* miR-30c*,* miR-30d*, and* miR-30e*. Downregulation of the* miR-30* family is observed in several types of cancer such as breast, bladder, and colon [26, 27]. Moreover, decreased expression of* miR-30* is observed in metastasis compared to the primary tumor [28], suggesting a role in aggressive disease. In ATC,* miR-30* expression is also reduced in tumor samples [20, 29]. Indeed, modulation of* miR-30* levels in ATC cells has a great impact in cancer cell biology. Importantly,* miR-30d* ectopic expression in an ATC cell line reduced monolayer cell growth and impaired anchorage-independent cell growth [30].

Downregulation of* miR-30* derepresses the expression of EZH2, an important component of the polycomb repressive complex 2 (PRC2) that regulates chromatin condensation and gene expression. EZH2 is the enzymatic subunit of histone methyltransferase that trimethylates histone H3 lysine 27. EZH2 is overexpressed in ATC and enhances cell proliferation, migration, and invasion, while repressing the expression of thyroid transcription factor PAX8 [31]. Another important cellular process regulated by the* miR-30* family is autophagy through targeting the key autophagy-promoting protein, Beclin1 (gene* BECN1*). In ATC,* miR-30d* restoration sensitizes cancer cells to cisplatin treatment by repressing Beclin1, which participates in the early stages of autophagosome formation [32]. An* miR-30d* mimic enhanced the apoptotic effects of cisplatin as shown by increased cleaved caspase-3 and PARP levels and annexin V staining [33]. Moreover, cisplatin treatment in a xenograft model also showed shrinkage of ATC tumor derived from* miR-30* overexpressing cells. The blockage of autophagy using specific inhibitors exerts similar effects to the reintroduction of* miR-30* into ATC cells, indicating that the autophagy process is important for ATC cells' resistance to chemotherapy.

### 2.3. *let-7* Family

Family genes of* let-7* are located on different chromosomes and are abundantly expressed in a normal thyroid gland (*let-7a*,* let-7b*,* let-7c*,* let-7d*,* let-7e*,* let-7f*, and* let-7g*) [34]. Deregulation of* let-7* is observed in several types of cancer, and its tumor suppressor effects are usually abolished by its downregulation [35].* let-7* was the first miRNA identified as having a role in cancer through validation of RAS protooncogene mRNA targeting and the association of low levels of* let-7* with a poor prognosis in lung cancer [36]. Downregulation of several members of the* let-7* family is observed in well-differentiated thyroid cancer (PTC and FTC) [37–39], but a marked decrease in the expression of* let-7a*,* let-7c*,* let-7d*,* let-7f*,* let-7g*, and* let-7i* is also observed in ATC [20, 21, 29].

Modulation of* let-7* levels alters thyroid cancer cell biology. Ectopic expression of* let-7f* in a PTC cell line inhibits cell proliferation and viability, while it reduces the activation of MAPK signaling, an important marker of thyroid cancer [40]. Moreover,* let-7* enhances the expression of thyroid transcription factor-1 (TTF1/NKX2-1), a key factor in maintaining the expression of iodine metabolizing genes and thyroid differentiation, usually lost in ATC. The recovery of* let-7a* expression in an FTC cell line changes cell morphology to an epithelial-like phenotype (flat and adherent), while it reduces cell migration by targeting* FXYD5*, an important regulator of cell adhesion [39]. Moreover, in aggressive lung cancer, loss of* let-7* is associated with a poorer prognosis [36], and the reduction of* let-7c* is associated with refractoriness to chemo- and radiotherapy treatments. Indeed, the ectopic expression of* let-7c* in a lung cancer cell line restores the cells' response to chemo- and radiotherapy and represses the EMT process [41], indicating an important role for* let-7* in tumor aggressiveness.

## 3. Upregulated miRNAs in ATC

Common miRNAs such as* miR-146*,* miR-221*,* miR-222*, and* miR-17-92* are upregulated in aggressive ATC and in well-differentiated thyroid cancer, indicating that reinforced expression of these miRNAs is important in maintaining the oncogenic process.

### 3.1. Cluster* miR-17-92*

The* miR-17-92* cluster is located on chromosome 13 and transcribes a polycistron that yields seven different mature miRNAs:* miR-17-5p*,* miR-17-3p*,* miR-18a*,* miR-19a*,* miR-19b*,* miR-20a*, and* miR-92a*. In normal thyroid follicular cells, early BRAF^V600E^ oncogene activation induces the expression of an* miR-17-92* cluster [42]. The BRAF oncogene is the most frequent genetic alteration in thyroid cancer (i.e., PTC) and is also detected in ATC, associated with poor clinical-pathological features of cancer such as extrathyroidal invasion, short time recurrence, and distant metastases [43, 44]. High levels of* miR-17-92* components are expressed in ATC [45], similar to that observed in other types of cancer, such as lung, colon, pancreatic, and lymphoma [46], especially in the aggressive forms of disease [47].

The molecular modulation of endogenous levels of these miRNAs using* LNA* (locked nucleic acid) modification resulted in important effects in ATC cell biology. Specific blockage of* miR-17-5p*,* miR-17-3p*, and* miR-19a* resulted in a pronounced growth inhibition of ATC cells and apoptosis through activation of caspase-3 and caspase-9 [45]. Inhibition of the* miR-17-92* cluster in ATC leads to the recovery of PTEN protein levels [45], an important negative regulator of PI3K growth signaling, which is repressed in ATC. Moreover, among several validated targets for the* miR-17-92* cluster are proteins associated with tumor aggressiveness. A key target that influences tumor invasion is TIMP-3, an important inhibitor of metalloproteinase activation, targeted by* miR-17-5p* and* miR-17-3p*.

### 3.2. *miR-146a* and* miR-146b*

The* miR-146* family,* miR-146a* and* miR-146b*, is overexpressed in ATC [18, 29, 48]. Despite sharing the same seed region, and therefore targets,* miR-146a* and* miR-146b* are transcribed by two independent genes located on chromosomes 5 and 10, respectively, and regulated by the transcription factor NF*κ*B. The promoter region of both miRNAs contains binding sites for the NF*κ*B complex, part of a key oncogenic signaling pathway, usually overactivated in ATC, which shows increased nuclear staining for RelA (p65), the subunit of the NF*κ*B dimer [49]. Ectopic expression of the inhibitory protein of this signaling, I*κ*B, decreases* miR-146a* and* miR-146b* levels in an ATC cell line [48]. Moreover, inhibition of* miR-146a* leads to an abrogation of anchorage-independent growth and invasion by ATC cells [48]. Interestingly, NF*κ*B activation is observed at the invasive front of aggressive PTC showing local invasion compared to the central region of the tumor [50] and also in response to a BRAF^V600E^ oncogene, leading to cell migration and invasion [51], and* miR-146b* upregulation [52]. Moreover, the introduction of* miR-146b* into PTC mutated cells (BRAF^V600E^ or RET/PTC1) enhances cell invasiveness and migration [53]. Therefore, the NF*κ*B signaling pathway and its transcriptionally activated miRNAs,* miR-146a* and* miR-146b*, play an important role in thyroid cancer aggressiveness and progression. Indeed, increased plasma circulating levels of* miR-146b* can be detected in papillary thyroid cancer before surgery, which also correlates with tumor aggressiveness and poor prognosis [54].

### 3.3. *miR-221* and* miR-222*

*miR-221/miR-222* is a cluster of miRNAs, located on chromosome X, which is deregulated in thyroid cancer. Although* miR-221* and* miR-222* overexpression is detected in differentiated (PTC and FTC) [17, 37] and anaplastic thyroid cancer cells [18, 29, 55], the expression of these miRNAs is associated with poor clinical-pathological features of cancer. In PTC and FTC, levels of* miR-221* and* miR-222* positively correlate with tumor aggressiveness, increased extrathyroidal invasion, tumor size, higher tumor node metastasis stage, and papillary thyroid cancer recurrence [18, 56–58]. Indeed, higher expression of* miR-221* and* miR-222* is present in metastatic, in comparison to nonmetastatic, cancers [59]. Moreover,* miR-222* increased circulating plasma level is associated with the presence of the BRAF mutation and recurrent papillary thyroid cancer [54].

Ectopic expression of* miR-221* in cancer cells results in a robust increase in anchorage-independent growth in soft-agar medium [37], pointing to a role for this cluster of miRNAs in the process of invasion and cell migration. Indeed, one target of the cluster is RECK, an inhibitor of metalloproteinase. Inhibition of* miR-221* impairs cell migration and invasion via upregulation of RECK while it reduces metastases in a colon cancer mouse model [60]. Both* miR-221* and* miR-222* also influence cell proliferation, once overexpression deregulates the cell cycle, by targeting the p27^kip1^ (CDKN1B) protein, a key regulator of cell cycle progression [61].

## 4. Concluding Remarks

Currently, there is no effective therapy to blunt the lethal course of ATC, therefore prompting trials of additional and innovative therapies for ATC. Molecular targeted therapy for ATC seems to be a promising approach to retard cancer growth and increase patient survival. The molecular modulation of miRNA levels using miRNA mimics or antimiRs, allied to a novel class of highly specific inhibitors of MAPK and PI3K signaling, for instance, may enhance the ATC response to conventional treatment [62].

Systemic miRNA injection has showed promising results using lipid and other carrier molecules for treating lung and prostate cancer in animal models. Intratumoral injection or tail vein injection of a lipid-based* miR-34a* inhibited orthotopic prostate cancer tumor growth and metastases in immune-deficient mice [63], and lentivirus mediated* miR-34a* delivery to prostate cancer cells completely inhibited tumor growth. Moreover, the growth of prostate cancer bone metastases was significantly inhibited by systemically injecting* miR-16* complexed with atelocollagen [64]. In lung cancer, systemic injection of a neutral lipid emulsion of* miR-34a* and* let-7* significantly decreased in tumor burden in a mouse model of non-small cell lung cancer (NSCLC) [65]. Interestingly, a promising drug called miravirsen (SPC3649) is under phase II clinical trial for treating hepatic cancer. Miravirsen is a 15-nucleotide locked nucleic acid-modified antisense oligonucleotide with high affinity and specificity to* miR-122*, an miRNA used by HCV for hepatic cells infection [66].

Further studies* in vitro* and in animal models concerning the functional role of miRNAs and the impact of modulating oncomiR endogenous levels in ATC are one key to their future application as therapeutic adjuvant treatments. In particular, ATC patients would benefit from the concomitant reintroduction of tumor suppressor miRNAs* miR-200* and* miR-30* and the inhibition of the oncogenic* miR-146*,* miR-221*/*miR-222*, and* miR-17-92* clusters, as these miRNAs target several deregulated processes such as cell growth, invasion, migration, and EMT.

## Figures and Tables

**Table 1:** Validated targets for deregulated miRNAs in ATC.

miRNAs		Validated targets			Cellular processes	References
Downregulated miRs	*miR-200 *family	ZEB1	ZEB2	*β*-Catenin	EMT and proliferation	[21, 67]
	* miR-30 *family	Beclin1	EZH2	VIM	Autophagy, chromatin condensation, and EMT	[30, 33, 67]
	*let-7 *family	RAS	HMGA2	LIN28	Proliferation, histone modification, and stemness	[67, 68]
	*miR-25 *	EZH2	BIM	KLF4	Chromatin condensation and apoptosis	[30, 67]
	*miR-125 *	MMP1	HMGA2	LIN28A	Invasion, histone modification, and stemness	[20, 67, 69]

Upregulated miRs	*miR-221/miR-222 *	p27	RECK	PTEN	Cell cycle, growth, and invasion	[20, 67]
	*miR-17-92 *cluster	p21	TIMP3	PTEN	Cell growth and invasion	[45, 67]
	*miR-146a/miR-146b *	NF*κ*B	THRB	SMAD4	Cell differentiation, proliferation, and invasion	[52, 67, 70, 71]
